# miR-541-3p Promoted Porcine Reproductive and Respiratory Syndrome Virus 2 (PRRSV-2) Replication by Targeting Interferon Regulatory Factor 7

**DOI:** 10.3390/v14010126

**Published:** 2022-01-11

**Authors:** Xibao Shi, Yuanhao Yang, Xiaozhuan Zhang, Xiaobo Chang, Jing Chen, Chao Wang, Aiping Wang, Jianhua Wang, Jianru Qin, Xianlong Ye, Wei Jin, Gaiping Zhang

**Affiliations:** 1 Center for Pathogen Research, Engineering Laboratory for Bioconversion Technology of Functional Microbes, College of Life Sciences, Henan Normal University, Xinxiang 453007, China; yangyuanhao3735@163.com (Y.Y.); zhangxiaozhuan0103@126.com (X.Z.); jh_wang@sibs.ac.cn (J.W.); 2019020@htu.edu.cn (J.Q.); yexianlong1988@163.com (X.Y.); jinwei@htu.edu.cn (W.J.); 2 Henan Provincial Key Laboratory of Animal Immunology, Henan Academy of Agricultural Sciences, Zhengzhou 450002, China; changxiaobo88@163.com (X.C.); y-y-c@163.com (C.W.); 3 International Joint Research Center of National Animal Immunology, Henan Engineering Laboratory of Animal Biological Products, College of Animal Science and Veterinary Medicine, Henan Agricultural University, Zhengzhou 450000, China; chenjing519@126.com; 4 Department of Bioengineering, Zhengzhou University, Zhengzhou 450000, China; pingaw@126.com

**Keywords:** PRRSV, type I interferon, miR-541-3p, IRF7

## Abstract

Porcine reproductive and respiratory syndrome (PRRS) is a disease caused by PRRS virus (PRRSV), which seriously harms the pig industry. Revealing the mechanism by which PRRSV inhibits immune response will help prevent and control PRRS. Here, we found that PRRSV-2 may hijack host miR-541-3p to inhibit host innate immune response. Firstly, this work showed that miR-541-3p mimics could facilitate the replication of PRRSV-2 and the results of the quantitative real time polymerase chain reaction (qRT-PCR) showed that PRRSV-2 could up-regulate the expression of miR-541-3p in MARC-145 cells. Since previous studies have shown that type I interferon could effectively inhibit the replication of PRRSV-2, the present work explored whether miR-541-3p regulated the expression of type I interferon and found that miR-541-3p could negatively regulate the transcription of type I interferon by targeting interferon regulatory factor 7 (IRF7). More importantly, PRRSV-2 infection could down-regulate the expression of IRF7 and over-expression of IRF7 could down-regulate the replication of PRRSV-2 in MARC-145 cells. In conclusion, PRRSV-2 infection up-regulated the expression of miR-541-3p to promote its replication in MARC-145 cells, since miR-541-3p can negatively regulate the transcription of type I interferon by targeting IRF7.

## 1. Introduction

Porcine reproductive and respiratory syndrome disease (PRRS), which is caused by PRRS virus (PRRSV) and is mainly characterized by reproductive failure of sows, high mortality of piglets and respiratory distress of pigs of all ages, is one of the most important diseases that seriously harm the pig industry. Taking the United States as an example, PRRS causes approximately USD 664 million in economic losses each year [1]. PRRSV contains a single positive-stranded RNA genome and belongs to the *Arteritis* virus family [2]. PRRS was first reported in the United States in the 1980s. Then, two genetically distinct PRRSV strains were isolated and have been divided into two independent species: PRRSV-1 (*betaarteryvirus suid* 1) and PRRSV-2 (*betaarteryvirus suid* 2) [3,4]. Both species have now spread around the world, but PRRSV-1 is prevalent mainly in Europe, while PRRSV-2 is prevalent in the Americas and Asia. PRRSV-1 is divided into four subtypes, and PRRSV-2 is divided into nine lineages. Due to the high frequency mutation of PRRSV gene, the gene recombination of different pedigrees/sublineages and the immunosuppression caused by PRRSV, there is still no effective vaccine to prevent PRRS. Therefore, revealing the mechanism of immunosuppression caused by PRRSV will provide help for the prevention and control of PRRS [5,6].

MicroRNAs (miRNAs) are small endogenous non-coding RNA molecules (21–22 nt) and take an important role in regulating almost all cellular functions such as cell differentiation, apoptosis, proliferation and carcinogenesis, since miRNA could degrade target mRNAs or prevent the translation of target mRNAs if the nucleotides 2–8 (seed sequence) of the miRNAs could bind to the 3′-untranslated region (3′-UTR) of the corresponding target mRNAs (5′-UTR of a few target mRNAs) [7,8]. Because one miRNA could target many mRNAs [9,10,11], miRNAs are also important molecules in the regulation of immune response. Other and our previous studies have shown that PRRSV infection has caused changes in miRNA expression profiles in MARC-145 cells and porcine alveolar macrophages (PAMs) and some PRRSV-regulated miRNAs have taken part in regulating the replication of PRRSV [12,13,14,15]. miR-23 [16], which is down-regulated by infection with PRRSV-2, could inhibit the replication of PRRSV since miR-23 positively regulates the transcription of type I interferon, while miR-373 [15], miR-382-5p [17] and miR-30c [18], which are up-regulated by PRRSV-2 infection, could facilitate the replication of PRRSV-2 since both miR-373 and miR-382-5p negatively regulate the transcription of type I interferon while miR-30c could negatively influence the type I interferon response. Our previous results of high-throughput sequencing of small RNAs from PRRSV-2-infected MARC-145 cells have shown that PRRSV-2 infection changes the expression profile of host miRNAs [15]. So, whether PRRSV regulates host miRNAs to modulate cellular immune response has attracted our attention. In this study, we selected several miRNAs which were regulated by PRRSV-2 infection to screen new miRNAs that could promote the replication of PRRSV-2. We found that miR-541-3p could promote the replication of PRRSV-2 since miR-541-3p could negatively regulate the transcription of type I interferon.

## 2. Materials and Methods

### 2.1. Cells and Virus

MARC-145 cells [19], which were derived from the African green monkey kidney cell line MA-104 and could be cultured in Dulbecco’s modified Eagle’s medium (DMEM) medium containing 10% fetal bovine serum (FBS), were susceptible to PRRSV-2 and were used in vitro studies of PRRSV-2. HEK 293T cells (ATCC ACS-4500), which were derived from the human kidney cell line HEK293 and were optimal for transient transfection and protein expression, were used in identifying the target genes of microRNA. Both of the above cells were incubated at 37 °C in an incubator with 5% CO_2_. PRRSV-2 strain BJ-4 (GenBank accession No. AF331831.1), a kind gift from Prof. Hanchun Yang (China Agricultural University, Beijing, China), was used in the present work.

### 2.2. Antibodies and Reagents

Antibody against IRF7 was purchased from Cell Signaling Technology (CST) (Beverly, MA, USA). Mouse anti-Flag antibody was purchased from Sigma-Aldrich (St. Louis, MO, USA). Mouse anti-glyceraldehyde 3-phosphate dehydrogenase (GAPDH) monoclonal antibody and anti-β-actin antibody were purchased from GenScript (Piscataway, NJ, USA). Horseradish peroxidase (HRP)-conjugated labeled Goat anti-mouse IgG secondary antibody was purchased from Jackson Immuno Research (West Grove, PA, USA). miR-541-3p mimics and inhibitors were purchased from Qiagen (Valencia, CA, USA). Polyinosinic-polycytidylic acid (poly(I:C)) was purchased from Sigma-Aldrich (St. Louis, MO, USA). Lipofectamine 2000 transfection reagent was purchased from Invitrogen (Carlsbad, CA, USA).

### 2.3. Plasmids

To construct the plasmid pcDNA6.2-miR-541-3p which could express miR-541-3p, the top strand oligo for miR-541-3p and bottom strand oligo for miR-541-3p in Table 1 were used for touchdown polymerase chain reaction (PCR), and then the primers pcDNA6.2-miR-541-3p-For and pcDNA6.2-miR-541-3p-Rev were used for production of the double-stranded oligo which contained the sequence of the pre-miR-541-3p, and finally the double-stranded oligo was cloned into vector pcDNA6.2 (Invitrogen) (Carlsbad, CA, USA) by using the BamHⅠ and BglII restriction sites. The IRF7-3′-UTR sequence which could be complementary to seed sequence of miR-541-3p was synthesized by Shanghai Shenggong Co., Ltd. (Shanghai, China) and then the double-stranded DNA sequence of IRF7-3′-UTR was formed by gradient cooling and annealing, and finally was cloned into vector psiCHECK-2 (Promega) (Madison, WA, USA) by using the XhoI and NotI restriction sites. The cDNA encoding amino acids of IRF7 was amplified by PCR and cloned into the HindIII and BamHI sites of the expression vector pCMV-Flag (IRF7-Flag). All primers and oligonucleotides used in this work were shown in Table 1. The p-284 Luc, which was an IFN-β promoter reporter plasmid, was constructed as previously described [20]. pRL-TK (Promega) (Carlsbad, CA, USA), which contained a Renilla-luciferase reporter gene, was used as an internal control in the dual luciferase reporter assay system.

### 2.4. RNA Quantification

The indicated cellular total RNA was extracted by the RNeasy Mini Kit (Qiagen) (Valencia, CA, USA) and was reverse transcribed by PrimeScript™ RT Master Mix (Perfect Real Time) (Takara) (Shiga, Japan) according to the manufacturer’s protocol. Then, quantitative real time PCR (qRT-PCR) was performed by using SYBR Green Master Kit (Takata) (Shiga, Japan) on a 7500 fast qRT-PCR system (Applied Biosystems) (Foster City, CA, USA). Primers are shown in Table 1. The results of qRT-PCR were analyzed by 2^−ΔΔ^*^C^*_T_ method with GAPDH as the internal reference [21]. In addition, miRNA analysis kits (Qiagen) (Valencia, CA, USA) were used to analyze the expression of the miR-541-3p according to the manufacturer’s instructions, while the U6 snRNAs were in place of GAPDH as the internal control in the method of 2^−ΔΔ^*^C^*_T_ for analyzing the relative expression levels of miRNA.

### 2.5. Dual Luciferase Reporter Assay

The indicated cells in 24-well plates were transfected with miR-541-3p mimics, miR-541-3p inhibitors or indicated control and p-284, RL-TK by using Lipofectamine 2000 transfection reagent. Twenty-four hours later, the cells were transfected with poly(I:C) (10 µg/mL (Sigma-Aldrich, St. Louis, MO, USA)). Then, 9 h later, the cells were harvested for luciferase activity assay by using the dual-luciferase reporter assay system (Promega) (Madison, WA, USA).

In order to identify the target mRNA of miR-541-3p, the HEK 293T cells were prepared in 24-well plates and then the cells were transfected with the pcDNA6.2-miR-541-3p or pcDNA6.2-Mut-miR-541-3p and psiCHECK-2-IRF7-3′-UTR or psiCHECK-2-Mut-IRF7-3′-UTR, and, 24 h later, the cells were harvested for luciferase activity assay by using the dual-luciferase reporter assay system.

### 2.6. RNA Interference Assay

The specific siRNAs to IRF7 and the control siRNAs (siNC) used in this experiment were synthesized by GenePharma (Shanghai, China), and the sequences of siRNAs are shown in Table 1. MARC-145 cells were prepared in 24-well plates and were transfected with 50 nM siRNA by using Lipofectamine RNAiMAX transfection reagent according to the manufacturer’s instruction. Next, 24 h later, the cells were infected with PRRSV-2 at a multiplicity of infection (MOI) of 0.1. Then, 2 h later, the cells were cultured with the fresh DMEM which contained 2% FBS. Finally, the cells were harvested at the indicated time for further analysis.

### 2.7. Virus Titration

Generally, PRRSV-2 infection could cause the cytopathic effect (CPE) of MARC-145 cells. The 50% tissue culture infected dose (TCID_50_) of the virus could be measured by observing the CPE, and then the TCID_50_ could be used to calculate the titer of the virus. So, the virus titration of PRRSV-2 was performed by using MARC-145 cells as previously described [22]. Briefly, MARC-145 cells were prepared in 96-well plates and were infected with ten-fold serial dilution of the indicated virus samples. Then, 1 h later, the supernatants were replaced with fresh DMEM which contained 2% FBS. Finally, 48 h (or the indicated time) later, the PRRSV titers were analyzed by theTCID_50_ according to the Reed–Muench method [23].

### 2.8. Statistical Analysis

Bar graphs were plotted to show the mean ± standard deviation (SD). All statistical analyses were performed by two-sided Student’s *t* test. All experiments were repeated in at least three independent experiments. *p* < 0.05 could be considered statistically significant and *p* < 0.01 could be considered highly significant.

## 3. Results

### 3.1. miR-541-3p Facilitated the Replication of PRRSV-2 in MARC-145 Cells

**Figure 1:**
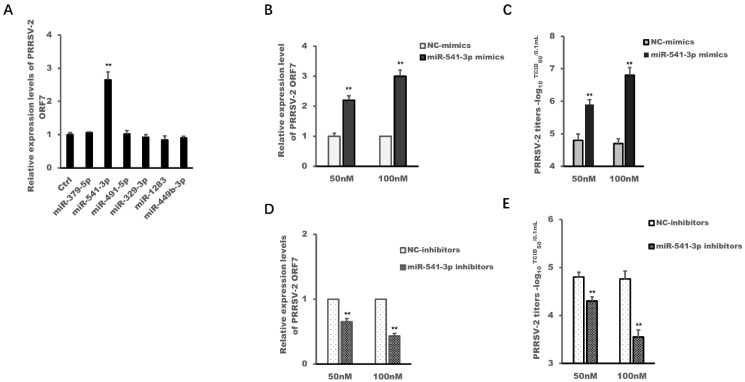
miR-541-3p promoted the replication of PRRSV-2 in MARC-145 cells. (**A**) MARC-145 cells were transfected with miR-379-5p mimics, miR-541-3p mimics, miR-491-5p mimics, miR-329-3p mimics, miR-1283 mimics or miR-449b-3p mimics, and 24 h later, the cells were infected with PRRSV-2 at a multiplicity of infection (MOI) of 0.1. Twenty-four hours later, the cells were collected for the analysis of RNA levels of Open reading frame 7 (ORF7) of PRRSV-2 by quantitative real time polymerase chain reaction (qRT-PCR). MARC-145 cells were transfected with miR-382-5p mimics (**B**,**C**), miR-382-5p inhibitors (**D**,**E**), negative control mimics (NC-mimics) or negative control inhibitors (NC-inhibitors) at a final concentration of 50 nM or 100 nM, and 24 h later, the cells were infected with PRRSV at an MOI of 0.1. Twenty-four hours later, the cells were harvested for the analysis of RNA levels of PRRSV-2 by qRT-PCR (**B**,**D**) and for the analysis of the PRRSV-2 titers by 50% tissue culture infected dose (TCID_50_) (**C**,**E**). Data are presented as the mean + standard deviations (SD). Student’s *t* test was used for statistical analysis. All experiments were repeated at least three times with similar results. ** *p* < 0.01.

Our previous results of high-throughput sequencing of small RNAs from PRRSV-2-infected MARC-145 cells showed that PRRSV-2 infection has changed the expression profile of host miRNAs, and both PRRSV-2-up-regulated miR-373 and PRRSV-2-up-regulated miR-382-5p could promote the replication of PRRSV-2 [15,17]. Therefore, the current work selected six miRNAs which were regulated by PRRSV-2 infection in MARC-145 cells, and used their mimics to explore whether these miRNAs regulated the replication of PRRSV-2. The results in Figure 1A showed that only miR-541-3p mimics could promote the replication of PRRSV-2, while miR-379-5p mimics, miR-491-5p mimics, miR-329-3p mimics, miR-1283 mimics and miR-449b-3p mimics could not.

To further confirm the above results, different concentrations of miR-541-3p mimics and miR-541-3p inhibitors were introduced in the next experiment. The results in Figure 1B,C show that the replication efficiency of PRRSV-2 in MARC-145 cells became higher and higher with the increase concentration of miR-541-3p mimics, while with the increase of the concentration of miR-541-3p inhibitors, the replication efficiency of PRRSV-2 in MARC-145 cells became lower and lower (Figure 1D,E). This meant that miR-541-3p mimics up-regulated the ORF7 expression levels of PRRSV-2 and the viral titers in a concentration-dependent manner in MARC-145 cells, while miR-541-3p inhibitors down-regulated the ORF7 expression levels of PRRSV-2 and the viral titers in a concentration-dependent manner too, which indicted that miR-541-3p facilitated the replication of PRRSV-2 in MARC-145 cells.

### 3.2. PRRSV-2 Infection Up-Regulated the Expression of miR-541-3p in MARC-145 Cells

**Figure 2:**
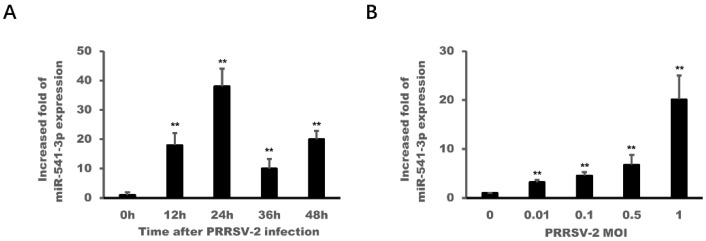
PRRSV-2 infection up-regulated the expression of miR-541-3p in MARC-145 cells. (**A**) MARC-145 cells were infected with PRRSV-2 (multiplicity of infection (MOI) = 1) for indicated times. Then, the expression levels of miR-541-3p were determined by quantitative real time polymerase chain reaction (qRT-PCR). (**B**) MARC-145 cells were infected with PRRSV-2 at different MOIs, and 24 h later, the expression levels of miR-541-3p were measured by qRT-PCR. All experiments were repeated at least three times with similar results. ** *p* < 0.01.

Our previous results of high-throughput sequencing of small RNAs have shown that PRRSV-2 infection down-regulated the expression of miR-541-3p [15], which was not confirmed by qRT-PCR, so a PRRSV-2-infected time-course experiment and a virus dose-dependent experiment were carried out in the present work and the expression levels of miR-541-3p were detected by qRT-PCR. The results in Figure 2 showed that the expression levels of miR-541-3p were up-regulated in MARC-145 cells with PRRSV-2 infection and reached a peak at 24 h post-infection (Figure 2A). The higher dose of PRRSV-2 could induce higher expression levels of miR-541-3p (Figure 2B). Above results indicated that PRRSV-2 infection up-regulated the expression of miR-541-3p in MARC-145 cells, which was not consistent with our previous results [15]. Considering the accuracy of fluorescent quantitative PCR and the rigor of time dynamic and dose gradient experiments, the result of the present work that PRRSV-2 infection up-regulated the expression of miR-541-3p was more reliable.

### 3.3. miR-541-3p Negatively Regulated Poly(I:C)-Induced Transcription of Type I Interferon

Now that miR-541-3p facilitated the replication of PRRSV-2, and PRRSV-2 infection up-regulated the expression of miR-541-3p in MARC-145 cells, the mechanism where miR-541-3p facilitates the replication of PRRSV-2 was explored in further experiments.

**Figure 3:**
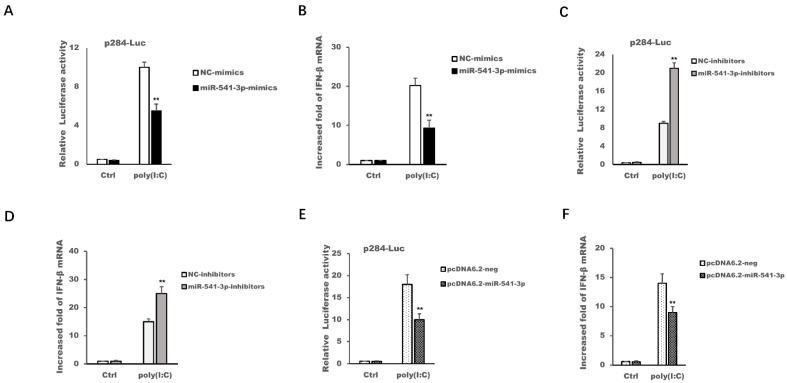
miR-541-3p inhibited poly(I:C)-induced production of type I interferon. MARC-145 cells were transfected with p-284, pRL-TK and miR-541-3p mimics (**A**), miR-541-3p inhibitors (**C**) or pcDNA6.2-miR-541-3p (**E**), and 36 h later, the cells were transfected with poly(I:C) (10 µg/mL), and 9 h later, the IFN-β luciferase activities were assayed by using the dual-luciferase reporter assay. MARC-145 cells were transfected with miR-541-3p mimics (**B**), miR-541-3p inhibitors (**D**) or pcDNA6.2-miR-541-3p (**F**), and 36 h later, the cells were transfected with poly(I:C) (10 µg/mL), and 9 h later, the mRNA expression levels of IFN-β were assayed by quantitative real time polymerase chain reaction (qRT-PCR). Ctrl indicated that the cells were not transfected with poly(I:C). All experiments were repeated at least three times with similar results. ** *p* < 0.01.

Other and our previous studies have shown that recombinant type I interferon could effectively inhibit the replication of PRRSV-2, and PRRSV-2 has evolved some mechanisms to antagonize the transcription of type I interferon [20,24,25,26,27], so the present work explored whether the PRRSV-2-up-regulated miR-541-3p modulated the transcription of type I interferon. The results in Figure 3 showed that miR-541-3p mimics and the expression plasmid of miR-541-3p could inhibit poly(I:C)-induced IFN-β promoter activation (Figure 3A,E) and could inhibit poly(I:C)-induced expression of IFN-β mRNA (Figure 3B,F), while miR-541-3p inhibitors could up-regulate poly(I:C)-induced FN-β promoter activation (Figure 3C) and could up-regulate poly(I:C)-induced expression of IFN-β mRNA (Figure 3D) in MARC-145 cells. These results indicated that miR-541-3p was a novel microRNA that negatively regulated the transcription of type I interferon.

### 3.4. Interferon Regulatory Factor (IRF) 7 Was a Target Gene of miR-541-3p

**Figure 4:**
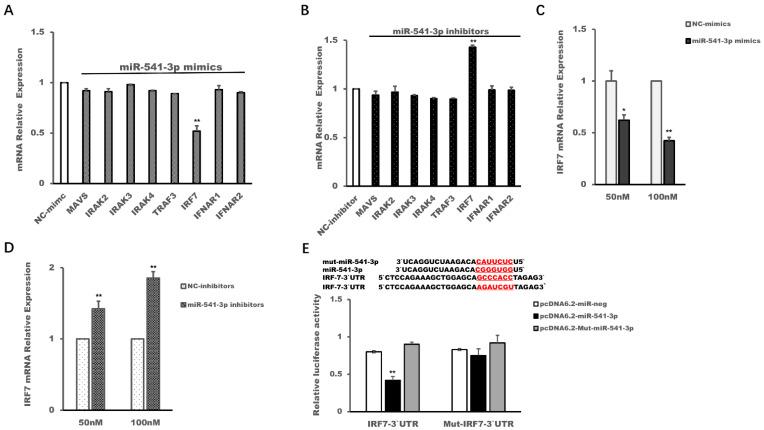
Interferon regulatory factor 7 (IRF7) was a target gene of miR-541-3p. HEK 293T cells were transfected with miR-541-3p mimics (**A**), miR-541-3p inhibitors (**B**), NC-mimics or NC-inhibitors. Then, 48 h later, the expression of predicted target mRNAs of miR-541-3p was measured by quantitative real time polymerase chain reaction (qRT-PCR). HEK 293T cells were transfected with miR-541-3p mimics at a final concentration of 50 nM or 100 nM (**C**), miR-541-3p inhibitors at a final concentration of 50 nM or 100 nM (**D**) and NC-mimics or NC-inhibitors. Then, 48 h later, the expression levels of IRF7 were measured by qRT-PCR. (**E**) Schematic presentation of base pairing between the 3′ untranslated region (UTR) of IRF-7 and the miR-541-3p sequence; the underlined red bases are their paired bases and their corresponding mutant bases. HEK 293T cells were transfected with the pcDNA6.2-miR-541-3p or pcDNA6.2-Mut-miR-541-3p and psiCHECK-2-IRF7-3′-UTR or psiCHECK-2-Mut-IRF7-3′-UTR, and 24 h later, the cells were harvested for luciferase activity assay by using the dual-luciferase reporter assay system. All experiments were repeated at least three times with similar results. * *p* < 0.05; ** *p* < 0.01.

It has been documented that miRNA regulates the physiological functions of cells by targeting its target mRNAs. So, in this study, TargetScan, PicTar and miRPathDB software was used to predict the potential target mRNAs of miR-541-3p. Finally, we selected mitochondrial antiviral-signaling protein (MAVS), interleukin-1 receptor-associated kinase-like (IRAK) 2, IRAK3, IRAK4, TNF receptor-associated factor (TRAF) 3, interferon regulatory factor (IRF) 3, interferon alpha/beta receptor (IFNAR) 1 and IFNAR 2 as candidate target genes for further verification by qRT-PCR in HEK 293T cells, since these genes were related to the transcription and function of type I interferon. The results in Figure 4 showed that miR-541-3p mimics could down-regulate the expression of IRF7 (Figure 4A) while miR-541-3p inhibitors could up-regulate the expression of IRF7 (Figure 4B). The concentration gradient experiments of miR-541-3p mimics and miR-541-3p inhibitors also confirmed the above results (Figure 4C,D).

Since miRNA made its target mRNA degrade or prevented the translation of its target mRNA by using the seed sequence (usually 6 to 8 bases) of miRNA to bind to the 3′-UTR (5′-UTR of a few target genes) of the corresponding target mRNA [7,8], we constructed the 3′-UTR of IRF7 report plasmid (psiCHECK-2-IRF7-3′-UTR), 3′-UTR mutant of IRF7 report plasmid (psiCHECK-2-Mut-IRF7-3′-UTR), miR-541-3p expression plasmid (pcDNA6.2-miR-541-3p) and seed sequence mutant expression plasmid of miR-541-3p (pcDNA6.2-Mut-miR-541-3p) to confirm whether IRF7 was the target mRNA of miR-541-3p. The results in Figure 4E showed that pcDNA6.2-miR-541-3p could down-regulate the relative luciferase activity of psiCHECK-2-IRF7-3′-UTR but could not down-regulate the relative luciferase activity of psiCHECK-2-Mut-IRF7-3′-UTR, so our present work indicated that IRF7 was the target mRNA of miR-541-3p, which was consistent with previous reports that IRF7 was the target mRNA of miR-541-3p [28].

### 3.5. PRRSV-2 Infection Down-Regulated the Expression of IRF7 and the Over-Expression of IRF7 Could Inhibit the Replication of PRRSV-2 in MARC-145 Cells

**Figure 5:**
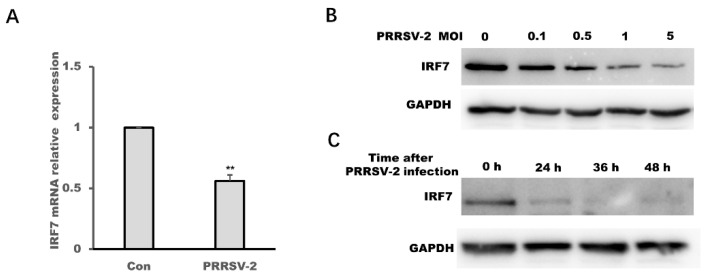
PRRSV-2 infection down-regulated the expression of interferon regulatory factor 7 (IRF7). (**A**) MARC-145 cells were infected with PRRSV-2 at a multiplicity of infection (MOI) of 0.1, and 36 h later, the mRNA expression levels of IRF7 were assessed by quantitative real time polymerase chain reaction (qRT-PCR). (**B**) MARC-145 cells were infected with PRRSV-2 at different MOI, and 24 h later, the protein expression levels of IRF7 were assessed by Western blots. (**C**) MARC-145 cells were infected with PRRSV-2 at an MOI of 0.1, and 24 h, 36 h or 48 h later, the protein expression levels of IRF7 were assessed by Western blots. All experiments were repeated at least three times with similar results. ** *p* < 0.01.

PRRSV-2 infection could up-regulate the expression of miR-541-3p and IRF7 was the target mRNA of miR-541-3p, so it is reasonable that PRRSV-2 infection could down-regulate the expression of IRF7. The results of qRT-PCR in Figure 5A show that the infection of PRRSV-2 could down-regulate the mRNA expression levels of IRF7 in MARC-145 cells. To confirm the above results, a virus dose-dependent experiment in Figure 5B and a time-course experiment in Figure 5C showed that PRRSV-2 infection also could down-regulate the protein levels of IRF7.

**Figure 6:**
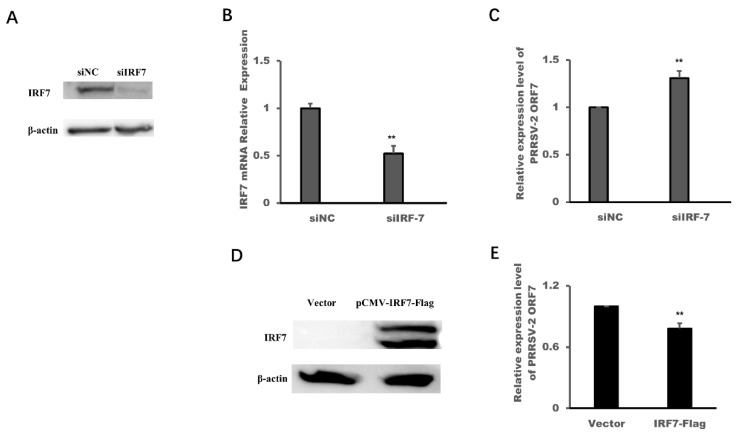
Interferon regulatory factor 7 (IRF7) inhibited the replication of PRRSV-2. MARC-145 cells were transfected with siIRF7 or siNC, and 48 h later, the protein levels (**A**) and the mRNA expression levels (**B**) of IRF7 were assessed by Western blots and quantitative real time polymerase chain reaction (qRT-PCR), respectively. (**C**) MARC-145 cells were transfected with siIRF7, and 24 h later, the cells were infected with PRRSV-2, and then the cells were harvested and the RNA levels of Open reading frame 7 (ORF7) of PRRSV-2 were determined by qRT-PCR. (**D**) MARC-145 cells were transfected with pCMV-IRF7-Flag, and 24 h later, the protein expression levels of IRF7 were assessed by Western blots. (**E**) MARC-145 cells were transfected with pCMV-IRF7-Flag, and 24 h later, the cells were infected with PRRSV-2, and then the cells were harvested and the RNA levels of PRRSV-2 ORF7 were determined by qRT-PCR. All experiments were repeated at least three times with similar results. ** *p* < 0.01.

Finally, we designed the siRNA of IRF7 to down-regulate the expression of IRF7 (Figure 6A,B) and constructed the expression plasmid of IRF7 to over-express IRF7 (Figure 6D) in MARC-145 cells. The results in Figure 6C showed that the expression levels of PRRSV-2 ORF7 in MARC-145 cells which were transfected with IRF7-siRNA(siIRF7) were higher than that in MARC-145 cells which were transfected with negative control siRNA. Over-expression of IRF7 could down-regulate the expression levels of PRRSV-2 ORF7 (Figure 6E), which indicated that IRF7 could inhibit the replication of PRRSV-2 but PRRSV-2 hijacked miR-541-3p to down-regulate the expression of IRF7 in MARC-145 cells.

## 4. Discussion

A virus is a strict parasitic pathogen. How to avoid the elimination of host cells is very important for the survival of the virus. In order to avoid the elimination of the host, a virus often uses its own components or the host components to regulate host physiological activities or host natural immunity. Therefore, identification of viral components or cellular components that could facilitate or inhibit virus replication is very important for the prevention and control of the virus.

PRRSV can cause persistent infection in pigs for more than 150 days [29]. Other and our previous studies have proved that PRRSV-2 can use its own components, such as non-structural protein 1α (nsp1α), nsp1β and nsp11, to inhibit inflammation response, the transcription of type I interferon and the innate immunity of RNAi [20,27,30,31]. Studies have also found that PRRSV-2 can change the expression profiles of host miRNA and PRRSV-2 used its up-regulated miR-373 and miR-382-5p to promote its replication since both of them can inhibit the transcription of type I interferon [15,17,18]. So, whether PRRSV-2 regulates host miRNAs for its replication has attracted our interest. The present work has obtained a convincing result that PRRSV-2 could up-regulate host miR-541-3p to facilitate the replication of PRRSV-2 in MARC-145 cells (Figure 1 and Figure 2).

Some studies have shown that miR-541-3p might act as a suppressor gene in hepatocellular carcinoma, non-small cell lung cancer and prostate cancer since miR-541-3p could target transmembrane protease serines 4 (TMPRSS4), transforming growth factor-β-induced factor 2 (TGIF2) and heat shock protein 27 (HSP27), respectively [32,33,34]. Although a study has shown that HIV infection can up-regulate the expression of miR-541-3p, it has not been reported whether miR-541-3p influences virus replication and whether miR-541-3p influences the transcription of type I interferon. Therefore, our present work not only found for the first time that miR-541-3p could promote the replication of PRRSV-2, but also revealed its molecular mechanism whereby miR-541-3p facilitated the replication of PRRSV-2. Other and our previous studies have shown that PRRSV-2 modulates the production of type I interferon and the recombined type I interferon could inhibit PRRSV replication in vitro and in vivo [24,25,26], so this study explored whether miR-541-3p is involved in regulating the transcription of type I interferon and found that miR-541-3p is a novel miRNA that could negatively regulate the production of type I interferon (Figure 3). The present work also confirmed that IRF7, which was an important transcription factor for the transcription of type I interferon, was the target mRNA of miR-541-3p (Figure 4). Consistent with the results that PRRSV-2 infection up-regulates the expression of miR-541-3p and IRF7 was the target mRNA of miR-541-3p, PRRSV-2 infection can down-regulate the mRNA expression and the protein expression of IRF7 (Figure 5). Furthermore, over-expression of IRF7 in MARC-145 cells could inhibit the replication of PRRSV-2 while down-regulation of IRF7 by siIRF7 could facilitate the replication of PRRSV-2 (Figure 6), which indicated that IRF7 was also involved in antagonizing the replication of PRRSV-2. In addition, in this study, only monkey-derived MARC-145 cells were used to explore the effect of miR-541-3p on the replication of one strain of PRRSV-2. Considering that pigs are the natural hosts of PRRSV and there are two species of PRRSV, both of which have virulent and normal strains [2], it is necessary to study the effects of miR-541-3p on the replication of PRRSV-1 and different PRRSV-2 strains in vivo and in vitro in future.

In conclusion, our present work showed that the PRRSV-2-up-regulated miR-541-3p could facilitate the replication of PRRSV-2 since miR-541-3p could inhibit the transcription of type I interferon in MARC-145 cells, which indicated that PRRSV-2 could use the host component, miR-541-3p, to suppress the host antiviral immune response in MARC-145 cells. However, it is necessary to study the effects of miR-541-3p on the replication of PRRSV-1 and different PRRSV-2 strains in vivo and in vitro in further.

## Figures and Tables

**Table 1:** Primers or oligonucleotides used in plasmid construction and quantitative real time polymerase chain reaction (qRT-PCR).

Primers	Sequence (5′-3′)
MAVS-For	CTGCCTCACAGCAAGAGACCA
MAVS-Rev	GTAGACACAGGCCACTTCGTC
IRAK2-For	AGGCACGGGAAGCCATTCGT
IRAK2-Rev	AGCCCAGCACAGGTAAGACA
IRAK3-For	TTGGTCCTGGGCACAGAAA
IRAK3-Rev	AATAGCTCGACGATGCCCCA
IRAK4-For	TTGCTCAGGGTGCAGCTAAT
IRAK4-Rev	GTGCAAGGCCAAAGTCAGAT
TRAF3-For	CTCACAAATGCAGCGTCCAG
TRAF3-Rev	GCTCCACTCCTTCAGCAGGT
IRF7-For	GCAACGTGAGGGTGTGTCTT
IRF7-Rev	GCTCCATGAGCAAGCACTCAA
IFNAR1-For	TCGTTTACACCATTTCGCAAAGCTCAAA
IFNAR1-Rev	ACGATCCAAAGCCCACATGACACTATCT
IFNAR2-For	TGTCATTGAAGAGCAGTCAGAGGGGATT
IFNAR2-Rev	GTTGAAGGAGGGTGCATTTTAAGGGAGA
GAPDH-For	TGACAACAGCCTCAAGATCG
GAPDH-Rev	GTCTTCTGGGTGGCAGTGAT
ORF7-For	AAACCAGTCCAGAGGCAAGG
ORF7-Rev	GCAAACTAAACTCCACAGTGTAA
IFN-β-For	CTAGCACTGGCTGGAATGAGACT
IFN-β-Rev	GGCCTTCAGGTAATGCAGAATC
Flag-IRF7-For	CCCAAGCTTATGGCCTTGGCTCCTGAGAG
Flag-IRF7-Ror	CGCGGATCCCTAGACGGGCTGCTCCAGCTC
Top strand oligo for miR-541-3p	GGATCCTGGAGGCTTGCTGAAGGCTGTATGCTGTGGTGGGCACAGAATCTGGACTGTTTTGGCCACTGACTGAC
Bottom strand oligo for miR-541-3p	CAAAACCGGTGACTGACTGTCAGGTCTGACACGGGTGGTGTCCTGTGTTCCGGACAATGATCGTGAGTGTACCTTGTTTACCGGGTCTAGA
pcDNA6.2-miR-541-3p-For	GGATCCTGGAGGCTTGCTGAA
pcDNA6.2-miR-541-3p-Rev	AGATCTGGGCCATTTGTTCCATGT
Top strand oligo for mut-miR-541-3p	GGATCCTGGAGGCTTGCTGAAGGCTGTATGCTGTCTCTTACACAGAATCTGGACTGTTTTGGCCACTGACTGAC
Bottom strand oligo for mut-miR-541-3p	CAAAACCGGTGACTGACTGTCAGGTCTGACACATTCTCTGTCCTGTGTTCCGGACAATGATCGTGAGTGTACCTTGTTTACCGGGTCTAGA
IRF7-3′UTR For	TCGAACTCCAGAAAGCTGGAGCAGCCCACCTAGAGCTGGCCGC
IRF7-3′UTR Rev	GGCCGCGGCCAGCTCTAGGTGGGCTGCTCCAGCTTTCTGGAGT
Mut-IRF7-3′UTR	TCGAACTCCAGAAAGCTGGAGCAAGATCGTTAGAGCTGGCCGC
Mut-IRF7-3′UTR	GGCCGCGGCCAGCTCTAACGATCTTGCTCCAGCTTTCTGGAGT
siIRF7-sense	CCAUCUUUGACUUCAGAGUTT
siIRF7-antisense	ACUCUGAAGUCAAAGAUGGTT-3

## Data Availability

Exclude this statement since the study did not report any data.
